# The central role of gastrin in gastric cancer

**DOI:** 10.3389/fonc.2023.1176673

**Published:** 2023-10-24

**Authors:** Helge Waldum, Patricia Mjønes

**Affiliations:** ^1^ Department of Clinical and Molecular Medicine, Faculty of Medicine and Health Sciences, Norwegian University of Science and Technology, Trondheim, Norway; ^2^ Department of Pathology, St. Olav’s Hospital – Trondheim University Hospital, Trondheim, Norway

**Keywords:** gastric cancer, types of gastric cancer, neuroendocrine carcinoma, *Helicobacter pylori*, gastrin

## Abstract

The prevalence of gastric cancer has markedly declined, but due to the high mortality rates associated with gastric cancer, it is still a serious disease. The preferred classification of gastric cancer is according to Lauren into either the intestinal type, which has a glandular growth pattern, or the diffuse type, which does not have glandular structures. Both types have been classified as adenocarcinomas, with the latter type based on periodic acid–Schiff (PAS) positivity presumed to reflect mucin. However, the presence of mucin in the diffuse type, in contrast to neuroendocrine/enterochromaffin-like (ECL) cell markers, has not been confirmed by immunohistochemistry and *in situ* hybridization. The ECL cells are probably prone to becoming cancerous because they do not express E-cadherin. Gastric cancer is unique in that a bacterium, *Helicobacter pylori*, is thought to be its main cause. *H. pylori* predisposes infected individuals to cancer only after having caused oxyntic atrophy leading to gastric hypoacidity and hypergastrinemia. No single *H. pylori* factor has been convincingly proved to be carcinogenic. It is probable that gastrin is the pathogenetic factor for gastric cancer due to *H. pylori*, autoimmune gastritis, and long-term prolonged inhibition of gastric acid secretion. Hypergastrinemia induces ECL cell hyperplasia, which develops into neuroendocrine tumors (NETs) and then into neuroendocrine carcinomas in rodents, a sequence that has also been described in humans. During carcinogenesis, the tumor cells lose specific traits, requiring that sensitive methods be used to recognize their origin. Gastric cancer occurrence may hopefully be prevented by *H. pylori* eradication at a young age, and by the reduced use of inhibitors of acid secretion and use of a gastrin antagonist in those with previous long-term *H. pylori* infection and those with autoimmune gastritis.

## Introduction

Gastric cancer prevalence has shown a marked decline during the last few decades (1), but this cancer is still associated with a high mortality rate and causes many deaths (2). There are marked geographical differences in the prevalence of gastric cancer, with the highest being in East Asia (3). The dissimilarity in prevalence is unlikely to be due to genetic differences, since in East Asian individuals who have moved to a Western country, the cancer risk approaches that of the general population in the new country (4). Thus, the reason for these differences in prevalence must be due to exposure to a carcinogenic agent at childhood. The recognition of *Helicobacter pylori* as the main cause of gastritis (5), and shortly afterwards of gastric cancer (6), were important breakthroughs that have led to the increased prevention of this type of cancer. In this review we will concentrate on the classification, etiology, and pathogenesis of gastric cancer, with the hope of improving the prevention of this disease that is still the cause of many deaths. In particular, we will focus on the role of neuroendocrine cells, mainly the enterochromaffin-like (ECL) cells, in gastric cancer, and the role of gastrin in the pathogenesis of gastric cancers, especially those due to *H. pylori*. Thus, the role of dedifferentiation of mature cells *contra* stop of differentiation of stem cells will be discussed, in addition to whether or not hormones are complete carcinogens. Furthermore, the specificity of the staining methods used to classify gastric carcinomas will be focused on.

## Gastric cancer

### Classification of gastric carcinomas, macroscopic and microscopic

Gastric cancers can be classified on the basis of their macroscopical appearance, according to Borrmann (7), and type IV cancer shows diffuse thickening and stiffening of the affected wall, giving rise to loss of compliance and resulting in early satiety. This type is also called linitis plastica. The histologic classification, in accordance with Lauren’s criteria, differentiates between the intestinal type, which has a glandular growth pattern, and the diffuse type, which lacks such structures (8). Nevertheless, tumors classified as being of the diffuse type have also been accepted as adenocarcinomas due to periodic acid–Schiff (PAS) positivity, which has been thought to reflect mucin, a substance regarded as exclusively present in exocrine cells. However, PAS has affinity not only to mucin, but also to glycoproteins in general (9). By using immunohistochemistry and *in situ* hybridization we have, however, failed to detect mucin expression in cancer cells of the diffuse type, and therefore the argument in favor of classifying these tumors as adenocarcinomas is not supported. On the other hand, the expression of general neuroendocrine markers, such as chromogranin A, synaptophysin, and neuron-specific enolase, and the ECL cell marker histidine decarboxylase (HDC) has been shown repeatedly by immunohistochemistry in gastric carcinomas, especially in tumors of the diffuse type (10, 11). Moreover, chromogranin A was detected using immuno-electron microscopy in granules in the carcinoma cells of diffuse gastric carcinomas (11). Chromogranin A was also shown in gastric carcinoma cells by *in situ* hybridization (12). The Lauren classification seems to reflect biologically important differences, since a type of gastric cancer seldom or never changes into the other (8), and the decline in the frequency of gastric cancer seen during the last few decades is due to the decline in gastric cancers of the intestinal type, whereas the frequency of the diffuse type is unchanged (1). In fact, there have been reports describing an increase in the frequency of the diffuse type (13). Based upon our studies we have claimed that the diffuse type cancers may be neuroendocrine carcinomas originating from ECL cells (14), which also develop into gastric neuroendocrine tumors (NETs) (formerly called carcinoids) (15). Gastric cancers can also be classified as conventional or early-onset gastric cancers according to the individual’s age at diagnosis (i.e., if the patient is diagnosed when they are aged 45 years or under) since there are some clinical differences according to the individual’s age at occurrence (16).

### The etiology/pathogenesis of gastric cancers

Under this heading we will cover both etiology and pathogenesis. The distinction between these may sometimes seem unclear since the same factor can play both roles. Thus, inflammation and gastrin are successive pathogenetic factors in gastric cancer caused by *H. pylori*.

#### H. pylori

In the 1940s, an association between the risk of gastric cancer and reduced gastric acidity was recognized (17), and a decade later the importance of gastritis in gastric carcinogenesis was realized, as gastric cancer seldom occurred in a stomach without gastritis (18, 19). The description of *H. pylori* as the dominating cause of both gastritis (5) and gastric cancer (6) explained the association between gastritis and gastric cancer, and, since *H. pylori* predisposes infected individuals to gastric cancer only after having induced oxyntic atrophy (20), the connection between hypoacidity and gastric cancer was also understood.

*H. pylori* was the first bacterium to be accepted as causing cancer (type 1 carcinogen) (21). A search for the carcinogenic mechanism was initiated, looking both at the host and at differences between strains of the bacterium. In particular, the *H. pylori* virulence factors cag A and vac A were focused on. However, patients infected with *H. pylori* with or without these virulence factors can develop gastric cancer, and as early as in 2000 it was concluded that they represented unfilled promises since cag A and vac A were not as significant as previously thought (22). In reality, a huge number of *H. pylori* factors have been proposed to be carcinogenic without consistent results. However, it may be that strains of *H. pylori* could differ with respect to their ability to infect and cause inflammation in different parts of the gastric mucosa, and, thus, indirectly have different carcinogenic potential. Similarly, factors in patients developing gastric cancer secondary to *H. pylori* have been examined to detect any explanation for the susceptibility, but without success. Apparently, *H. pylori*-related gastric cancers did not show any specific genetic changes, and researchers have concluded that *H. pylori* could predispose infected individuals to gastric cancer indirectly (23). However, most individuals with *H. pylori* gastritis are infected during childhood (24), and reinfection after *H. pylori* eradication is seldom. The initial infection with *H. pylori* results in an acute phase, with dyspepsia and nausea (25), followed by a chronic phase. At this stage, the bacterium is confined to the antral mucosa and NH3 production because its urease activity may stimulate gastrin release, resulting in increased acid secretion and therefore predisposing to duodenal ulcer. A duodenal ulcer promoting gene, *dupA*, which is associated with duodenal ulcers and a reduced risk of gastric cancer, has also been described (26). However, even for this gene, there are overlaps, with duodenal ulcer occurring in individuals infected with *H. pylori* negative for dupA and cancer in some carrying *H. pylori* expressing it (26). With time the infection spreads to the oxyntic mucosa, which may lead to oxyntic atrophy, causing reduced gastric acid secretion and gastric hypoacidity, which necessarily causes hypergastrinemia. It has been established that eradication of *H. pylori* after oxyntic atrophy does not eliminate the risk of cancer development (27). Oxyntic atrophy and intestinal metaplasia are associated with risk of gastric cancer, although the latter is not necessarily a precursor (28–30). There have during the last few years been multiple reports of increased risk of gastric cancer after *H. pylori* eradication (31–34). The fact that the risk of cancer development continues after *H. pylori* eradication if oxyntic atrophy is present suggests that *H. pylori* itself is not carcinogenic, but that the inflammation it causes leads to atrophy of the acid, producing mucosa which predisposes individuals to cancer. Often in patients with advanced oxyntic atrophy, there is no more inflammation and *H. pylori* disappears, since there is no more acid to neutralize NH3 produced by the *H. pylori* urease. Thus, the inflammation does not seem to be the direct carcinogenic factor since the cancer risk continues. The persistent cancer risk after the loss of *H. pylori* may be due to the change in microbes due to hypoacidity or secondary to hypergastrinemia. Interestingly, the diffuse type of cancer seems to prevail among cancers that develop many years after *H. pylori* eradication (34). Hypergastrinemia, both with persisting and previous *H. pylori* infection, seems to be the most probable mechanism for the carcinogenic effect (35), like for autoimmune gastritis (36). *H. pylori* infection has been reported to cause 89% of global gastric cancers (37). Moreover, *H. pylori* infection also plays a dominant role in early-onset gastric cancers (38, 39). In contrast to gastric cancer, the other malignancy due to *H. pylori*, gastric mucosa-associated lymphoma tissue (MALT) lymphoma, seldom recurs after *H. pylori* eradication (40), indicating that they have a different pathogenesis. The very few relapses of gastric MALT lymphoma after *H. pylori* eradication may be due to the persistence of the lymphoma and may not represent a *de novo* development (41). The gastric MALT lymphomas are most probably the consequence of the continuous long-term stimulation of lymphocyte proliferation and are naturally not related to hypergastrinemia, since lymphocytes do not express the gastrin receptor or receptors for any of the ECL cell mediators (no lymphoma was detected in patients with gastrinoma). Interestingly, *Helicobacter suis* can infect humans and give rise to gastric MALT lymphoma (42), and may also induce gastritis, mimicking *H. pylori* gastritis (43).

### The pathogenesis of H. pylori and autoimmune gastritis in gastric cancer via hypergastrinemia

#### Gastrin and the target cell, the enterochromaffin-like (ECL) cell

Since the description of Zollinger–Ellison syndrome, it has been known that gastrin has a positive trophic effect on the oxyntic mucosa (44). Gastrin was long believed to have a direct effect on parietal cells, stimulating acid secretion directly (45), although studies of the regulation of acid secretion conducted in the mid-1970s using oxyntic mucosa in Ussing chambers (46) and studies on acid secretion in isolated glands (47) did not confirm this view. In the isolated rat stomach, maximal gastrin-stimulated acid secretion was inferior to maximal histamine-stimulated acid secretion, and gastrin, in contrast to a cholinergic drug, did not augment histamine-stimulated acid secretion (48). Moreover, gastrin stimulated histamine release (11), and a gastrin analog bound to the ECL cell and not to the parietal cell (49), all compatible with gastrin stimulating acid indirectly via the stimulation of histamine release from the ECL cell. The gastrin receptor was cloned from oxyntic mucosal cells enriched in parietal cells (50), a preparation that obviously must have contained ECL cells. It is still claimed that a gastrin receptor is localized on parietal cells, although not as being involved in the stimulation of gastric acid secretion, but rather as playing a role during a phase of parietal cell differentiation (51). Since gastrin shows the same trophic effect on parietal cells as other cells in oxyntic mucosa except ECL cells (52), this effect, if it is present, must be very weak.

**Figure 1:**
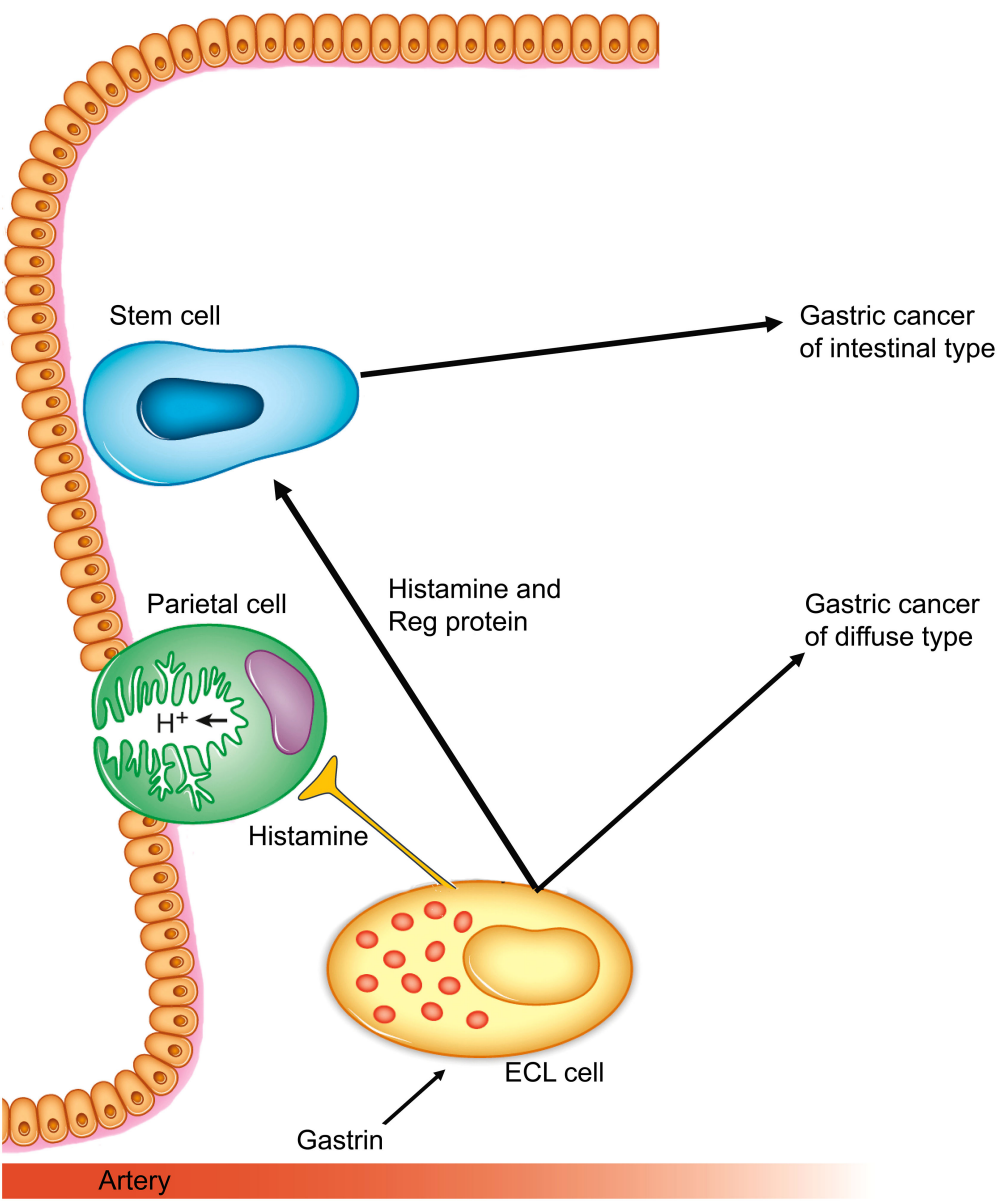
Gastrin and the ECL cell are central in gastric cancers of both diffuse and intestinal types. (Reproduced with permission from the publisher from Int J Mol Sci 2019; 20, doi: 10.3390/ijms20102444) (55).

Among the many neuroendocrine cells in the stomach, ECL cells are the most prevalent of those in the oxyntic glands. ECL cells were recognized as the cells producing the histamine that took part in the regulation of gastric acid secretion in rodents (53), and many years later also in humans (54). The central role of ECL cells in gastric physiology and pathology has previously been reviewed (55). ECL cells do not express E-cadherin (56), which probably contributes to the spreading liability of ECL cell-derived tumor cells. ECL cells produce basic fibroblast growth factor (57), which could be responsible for the fibrosis seen in patients with a gastric carcinoma of the diffuse type. Moreover, ECL cells also produce Reg protein, which has a stimulatory effect on gastric cell proliferation (58) and could be a factor in gastric neoplasia (59), and then mainly in gastric carcinomas of intestinal type (**Figure 1**). Interestingly, although ECL cells in mice do proliferate (60), this has not been shown in human participants, although in early phases of hypergastrinemia ECL cells were found to be grouped together as, for instance, linear or micronodular hyperplasia (61). Moreover, it is possible that specific traits are not expressed during phases of cell division. Interestingly, very recently it was reported that cells may lose contents before mitosis (62), which could be an explanation of the finding of no proliferation in gastric cancer cells expressing neuroendocrine markers (63). Furthermore, as we see it, it does not matter whether ECL cell precursors or mature ECL cells proliferate. It should also be noted that there is currently no convincing evidence of a gastrin receptor (CCK2) being present on stem cells, which could indicate that the general trophic effect of gastrin on the oxyntic mucosa is an indirect one on stem cells that is mediated by signal substances from the ECL cells.

The trophic effect of gastrin on the ECL and the risk of ECL tumors in humans after long-term hypergastrinemia was discovered in the first half of the 1980s (64). With the introduction of the potent inhibitors of gastric acid secretion belonging to the proton pump inhibitors, omeprazole, and the insurmountable histamine-2 antagonist loxtidine, it was also shown that in rodents long-term hypoacidity-induced ECL cell-derived tumors (65, 66), which were soon realized to be due to the trophic effect of gastrin (67). The potency of functional and trophic effects in rats (11) and in humans (68, 69) are quite similar and in agreement with interaction with the same receptor and showing that maximal trophic effect is reached at a rather low concentration. In rodents such as rats (65), mice (66), Japanese cotton rats (70), Mongolian gerbils (71), and Mastomys (72), gastrin induces gastric tumors of ECL cell origin. Moreover, the carcinogenic effect of gastrin was also demonstrated in the transgenic INS-GAS mice in which gastrin is over-expressed (73).

In humans, hypergastrinemia is associated with gastric tumors of low malignancy, that is, so-called neuroendocrine tumors (NETs), originating from ECL cells in patients with autoimmune gastritis (74), gastrinoma as part of multiple endocrine neoplasia type I (MEN I) (75), the sporadic gastrinoma not related to any syndrome (76, 77), or the long-term profound inhibition of acid secretion (55, 78, 79). The higher frequency of ECL cell NETs in patients with gastrinoma as part of MEN I than in those with sporadic gastrinoma has been attributed to the genetic defect affecting neuroendocrine cells, including ECL cells. However, there is a case report of gastrinomas combined with MEN I in which the gastrinomas were removed by surgery, resulting in the disappearance of the ECL cell NETs (80), suggesting that the difference in the frequency of ECL cell NETS in patients with sporadic gastrinomas and those in MEN I patients simply reflects the fact that the latter group had hypergastrinemia for a longer time. In autoimmune gastritis, it has been presumed that the inflammation predisposes individuals to the development of ECL cell NETs, and therefore these gastric NETs were subclassified as group 1, those due to gastrinoma were subclassified as group 2, and those showing normal gastrin levels were subclassified as group 3 (81). It should be noted that nobody has explained why the inflammation has a selectively trophic effect on ECL cells. With the increasing acceptance of proton pump inhibitor-induced gastric NETs, we (82) and others (83) have come up with a new way to classify these tumors. We suggested that group 1 should comprise gastric NETs due to hypergastrinemia and that group 2 should comprise those not showing hypergastrinemia (82), whereas Rais et al. suggested a new group for tumors occurring due to hypergastrinemia that are not related to oxyntic atrophy or gastrinoma (83). It should be noted that not only mutagens but also mitogens can induce tumors, since each cell division is accompanied by a low but definite risk of mutation. It is also probable that accelerated proliferation could increase the basal rate of mutations by reducing the time needed for gene repair.

Benign tumors may develop into more malignant ones by the accumulation of new mutations, as seen in gastric ECL cell NETs degenerating into carcinomas (84). Moreover, autoimmune gastritis predisposes affected individuals both to ECL cell NETs and to gastric carcinomas (85, 86). The carcinomas occurring in patients with pernicious anemia have been classified as adenocarcinomas, but by a thorough immunohistochemical examination of the cancers for neuroendocrine/ECL cell differentiation it was shown that most of these cancers expressed neuroendocrine/ECL cell markers (10). Thus, hypergastrinemia may drive the development of ECL cells, via phases of hyperplasia and NETs, to highly malignant tumors. The gastric malignant neoplasms found in patients with autoimmune gastritis have been classified as adenocarcinomas, but they may be neuroendocrine carcinomas (10). In fact, some years ago an ECL cell NET secondary to autoimmune gastritis was followed during the process of development to a highly malignant cancer which killed the patient (84). Moreover, only gastrin, and not any form of inflammation, can be the driver of the continuous process in the direction of further malignancy occurring in metastases. Accordingly, gastrin is a complete carcinogen for ECL cells. This has important clinical consequences. Since we do not know the etiology or pathogenesis of autoimmune gastritis nor have any treatment influencing its progression, the only way of reducing the gastric cancer risk in patients with autoimmune gastritis is endoscopic control until a gastrin antagonist becomes clinically available. In fact, since the 1990s there has existed a highly effective and specific gastrin antagonist, netazepide, with few, if any side effects (87, 88). In the mid-1990s the company owning netazepide conducted a survey, contacting 15 experts within the field(s) of gastric physiology/clinical gastroenterology about the potential of this compound. Only one expert (HW) responded positively; the others claimed that there was no need to investigate this compound since proton pump inhibitors (PPIs) covered the clinical need. Thus, the company sold the compound to the Sir James Black Foundation. Sir James Black recognized that netazepide was a promising compound, and he believed that it would be the third important new drug he would develop, after propranolol and cimetidine. Unfortunately, Sir James Black died before he could develop netazepide into a commercial drug. Due to our friendly relationship with Sir James Black, we got netazepide at an early phase and could show that it made gastric NETs disappear during treatment without causing any side effects (88). However, because of the peculiar reluctance of many experts to accept any risk of hypergastrinemia (89, 90), we have not had a clinically available gastrin antagonist for all these years. It is a pity that netazepide has not been available, since it could possibly be effective not only in gastric NETs but also gastric cancers which often express gastrin receptors (91). Although *H. pylori* gastritis and autoimmune gastritis induce oxyntic atrophy and predispose affected individuals to both ECL cell NETs (92, 93) and gastric carcinomas (6, 85, 94), autoimmune gastritis is more prone to develop into ECL cell NETs than *H. pylori* infection, whereas the opposite is true for gastric carcinomas (95). This may be explained by the duration and severity of atrophic gastritis, which starts later but becomes more rapidly severe in autoimmune gastritis than *H. pylori* gastritis (96). Gastric NETs due to hypergastrinemia are well known to occur in young people also, but hypergastrinemia due to autoimmune gastritis has not been described in early-onset gastric cancer, possibly due to the late onset of autoimmune gastritis. One article has described a lower risk of gastric cancer in patients with autoimmune gastritis than in non-autoimmune gastritis despite higher gastrin values in the former (97). This is most probably due to a different duration of hypergastrinemia, as autoimmune gastritis, as a rule, starts in adults. The level of hypergastrinemia also differs between the two conditions, which may play a role in the development of NETs *contra* cancers. Oxyntic atrophy secondary to autoimmune gastritis and *H. pylori* both give rise to intestinal metaplasia, which differs between the two causes with respect to proliferative lineages (98). Gastric intestinal metaplasia in most cases occurs in a mucosa with atrophy, and the prevalence of gastric cancer is reflected in the occurrence of gastric atrophy and intestinal metaplasia (99). Admittedly, there is no report describing gastric neoplasia as being more malignant than NET in patients with sporadic gastrinoma, which may be explained by the limited survival period for gastrinoma patients after diagnosis who have not been cured by surgery. It should also be noted that, until recently, normal fasting gastrin values have been overestimated, since at the time of developing gastrin immunoassays individuals with asymptomatic *H. pylori* gastritis with elevated gastrin levels (100) were included within control groups (101, 102). Moreover, particularly with reduced capacity to secrete acid, fasting gastrin values underestimate the gastrin values after meals (103). Accordingly, the 24-h gastrin exposure in persons with *H. pylori* gastritis is higher than fasting gastrin values indicate. In a very recently published paper, Rugge et al. tried to show that autoimmune gastritis *per se* did not induce gastric cancer since they found no gastric cancers in a group of patients with only oxyntic inflammation, but without or with only slight oxyntic atrophy (104). They claimed that *H. pylori* infection, although not detectable, was necessary to inducing gastric cancer in patients with autoimmune gastritis. This study failed to take into consideration that oxyntic atrophy is central not only to *H. pylori* gastritis (20) but probably also to autoimmune gastritis, and, even more importantly, that cancer development is a process that takes place over several years (105). Only elevated gastrin values of above 400 pg/mL (approximately 200 pmol/L) have been regarded as of importance in gastric carcinogenesis (90). However, gastrin is a potent hormone reaching near-maximal functional (68) and trophic (69) effects at this concentration. Moreover, there is no threshold concentration for the trophic effect of gastrin (106). Thus, the 24-h gastrin value multiplied with the time will determine the probability for gastric neoplasia. It is undisputed that *H. pylori* gastritis predisposes infected individuals to gastric carcinomas not only of the intestinal type, but also the diffuse type (20, 107, 108). There are many case reports describing a connection between carcinomas of the diffuse type of the signet ring cell (scirrhous) subtype and neuroendocrine expression (109–112). An even more recent study reported that germ line variants of genes known to predispose to gastric cancer, also affected cancer risk, and that *H. pylori* infection had an additive effect (113). However, there was no potentiating effect (113) which would have been expected if *H. pylori* had a direct effect via any of these genes.

For completeness, it should be noted that there are other types of gastric carcinomas classified as adenocarcinoma of fundic gland types and foveolar carcinomas occurring in non-*H. pylori*-infected subjects that are apparently not related to hypergastrinemia since they are also not related to autoimmune gastritis (114). There is only one case report that describes a malignant transformation of a gastric foveolar hyperplastic polyp in the context of autoimmune gastritis (115).

**Table 1** summarizes why the role of gastrin has hitherto been underestimated in gastric carcinogenesis.

**Table 1:** Gastrin has for decades been known to be an important trophic hormone for the oxyntic mucosa. Its role in gastric carcinogenesis has been underestimated because:.

Although gastrin was recognized as central in gastric NET development in humans (64) and rodents (65) from the mid-1980s, such tumors were not regarded as relevant to human gastric carcinomas because it was believed that human ECL cells could not transform into malignancies (116), Therefore, our group has mainly focused on neuroendocrine/ECL cell differentiation in human gastric cancers (55, 117, 118). However, a recent review by another group has focused on the central role of gastrin in gastric carcinogenesis (119).
Due to the inclusion of *H. pylori-*infected individuals in control groups, upper normal gastrin values have been overestimated (101, 102).
Due to the high affinity between gastrin and its receptor (stimulation of histamine release (120): with subsequent stimulation of acid secretion (68) and the trophic effect on the ECL cells (69, 121), sensitivity is high. Thus, the major trophic effect is reached at a concentration of approximately 100pmol/L (121), and trophic effects occur at gastrin concentrations previously thought to be in the normal range.
There is no threshold for the trophic effect of gastrin on ECL cells (106).
Fasting gastrin levels underestimate meal-induced gastrin especially in conditions with reduced levels of gastric acid secretion (103).
The tumorigenic effect depends on the 24-h level of gastrin (up to the concentration giving maximal effect, above which no further effect is obtained (69)) multiplied by the time of elevation.
It was found that *H. pylori* gastritis only induces slight hypergastrinemia before causing oxyntic atrophy, explaining the many years of latency between childhood infection and clinical gastric cancer. With oxyntic atrophy, gastrin is further increased (122) and hypergastrinemia predisposes affected individuals to carcinomas that mainly develop in the oxyntic mucosa (123).
Every condition with long-term hypergastrinemia [*H. pylori* gastritis (36), autoimmune gastritis (124), long-term use of drugs with profound inhibition of gastric acid secretion (122), and congenital defect H+/K+- ATPase (126)] predisposes affected individuals to gastric neoplasia.
Patients with sporadic gastrinoma develop ECL cell NETs (76, 77), but there is no report of any adenocarcinoma, which may be explained by there experiencing a too-short period of hypergastrinemia, since approximately 40% of patients are cured by surgery (126), and the period of hypergastrinemia required to induce more malignant tumors than ECL cell NETs is rather long (127).

#### Inflammation

The role of inflammation in carcinogenesis has gained more recognition during the last few decades. However, distinguishing between the effect of inflammation and the cause of inflammation may be difficult. As has already been stated, inflammation due to bacteria has not been shown to induce cancer before *H. pylori* infection, and even in this case it seems not to be a direct effect of the bacterium itself, but a consequence of the hormonal changes that the inflammation causes (127). The rarity of bacterial infections as the cause of cancer development may also be due to the fact that bacterial infections seldom become chronic and last for years. Viral infections, on the other hand, can persist for decades, causing chronic inflammation. For a gastroenterologist it is natural to mention hepatitis B (HBV) and hepatitis C (HCV) viruses. A few decades ago, no effective treatment for these two infections, which both predispose affected individuals to hepatocellular carcinomas, existed. However, we are now able to eradicate HCV and to suppress HBV. The eradication of HBV has not been successful due to the integration of its DNA genome into the host cell (128). HCV and, in particular, HBV have been accepted to be carcinogenic in and of themselves (129) and not only due to the inflammation they cause. However, inflammatory liver disease not due to virus infection also predisposes affected individuals to hepatocellular cancer (130). A possible mechanism may be the stimulation of proliferation secondary to the loss of hepatocytes due to inflammation. A similar mechanism may also be involved in gastric carcinogenesis secondary to gastritis. However, hypergastrinemia secondary to oxyntic atrophy is the probable mechanism for the carcinogenesis in autoimmune gastritis, in which one can follow the ECL cell changes via hyperplasia to dysplasia and neoplasia of the NET type, and then to neuroendocrine carcinomas (84). Interestingly, seropositivity not only for *H. pylori*, but also for autoimmune gastritis in young women was reported to predispose them to both early-onset and traditional-onset gastric cancers (131), reflecting the long latency for gastric cancer in general.

#### PPIs

Long-term PPI treatment induces the risk of gastric NETs (79, 82, 132) and also gastric cancer (33, 133–136), presumably via a reduction in gastric acidity and hypergastrinemia. In the 1940s it was discovered that there was a connection between hypoacidity and gastric cancer (17), and it was not surprising when in the mid-1980s the profound inhibition of gastric acid secretion by two different mechanisms was shown to give rise to malignant tumors in the oxyntic mucosa of rodents (65, 66). The tumors were identified as originating from ECL cells, and gastrin, as the main trophic hormone of ECL cells, was accepted as the cause (67). The tumorigenesis in the rodents passed through a phase of ECL cell hyperplasia via dysplasia to NETs. Whereas the histamine-2 blocker loxtidine was not developed into clinical use, the PPI omeprazole was accepted for use in severe hyper-acidic diseases such as gastrinoma and severe peptic ulcer disease. Within a short period, it was reported that omeprazole induced hypergastrinemia with accompanying ECL hyperplasia (137), that is, the same initial changes as those seen in rodents were observed. However, it was claimed that in humans this process would not develop further and that human ECL cells could not be transformed into malignant cells (116). Taking into consideration the rat experience (65), and that rats and humans are more than 90% similar genetically, this statement by Solcia et al. (116) seems peculiar. Moreover, the normal level of gastrin was set too high due to the inclusion of individuals with *H. pylori* gastritis within the control groups (101, 102) and values close to the maximal effective concentration (116) were set as level of risk (90). Last, but not least, observation periods of a few years were regarded as sufficient (138), not taking into consideration the great difference in life expectancy between rats and humans. As could have been expected, long-term PPI treatment has been reported to cause not only gastric NETs, but also carcinomas. The first publications were only case reports (133, 139), but in 2017–2018 came the first case-control study (33) and the first cohort study (140), both describing an increased risk of gastric carcinomas in patients treated with PPIs. In a retrospective study on patients in whom *H. pylori* had been eradicated, Niikura et al. confirmed that those patients treated with PPI after the eradication had an increased risk of gastric cancer (134). Later, many publications, including a meta-analysis (141), have appeared that describe a moderately increased risk of gastric cancer in patients using PPIs on a long-term basis. The increased risk seems to be small, but it must be remembered that carcinogenesis is a long-term process. In a very recently published paper with the title “Proton pump inhibitors and increased risk of gastric cancer: how much more evidence is needed?”, Brusselaers and Simin concluded that, currently, a significant proportion of gastric cancers in the Western world could be due to PPI use (142). Moreover, there is every reason to fear that in the future the scale of this problem will only increase. Due to their denial of the fact that PPIs can cause gastric cancer (143), many clinicians have not taken any notice or registered the use of PPIs in patients with gastric cancer. On the other hand, chronic gastrin hyperstimulation induces NETs tumors with a better prognosis, or at least one that is not worse, than for those that develop apparently spontaneously (81, 144). This most probably reflects the fact that a tumor developing with constant growth stimulation does not tend to undergo mutations that are as serious as those of tumors in which the growth changes are dependent only on mutations. In other words, macroscopic tumors secondary to chronic exposure to a trophic stimulus may appear at an earlier stage than those occurring without an obvious cause. In one study we found that gastric cancer accompanied with and not accompanied with hypergastrinemia were associated with similar mortality rates (145).

#### Infections

As previously stated, *H. pylori* is the main cause of gastric cancer (6). However, in recent years it has also been more focused on other microorganisms in the stomach and their role in gastric carcinogenesis (146). However, hitherto only Epstein–Barr virus (EBV) has been shown to be an important cause of gastric cancer among non-*H. pylori* microorganisms (147). Typically, Epstein-Barr virus gastric cancer has a dense lymphoid stroma, and may respond to immunotherapy (147). EBV-associated gastric cancers can be diagnosed by *in situ* hybridization, and in a large surgical study of gastric cancers 6.1% were positive (148).

#### Food

High salt intake has traditionally been claimed to increase the risk of gastric cancer. In a recent study conducted in China, however, salted fish, in contrast to processed meat, did not increase the risk of gastric cancer (149). Alternatively, then, it may be that meat not exposed to heat could contain microorganisms that have a carcinogenic effect, and thus that the effect is not related to salt. Microorganisms in fish, being far removed from us in the evolutionary sense, are probably associated with a lesser risk. The intake of fruit and vegetables has, on the other hand, been thought to reduce gastric cancer risk, although which factors have such an effect are not established (150). Excluding food and water contaminated with *H. pylori*, we will conclude that the intake of different types of food may have only a slight effect on gastric carcinogenesis.

#### Tobacco smoking

Tobacco smoking seems without doubt to predispose individuals to gastric cancer. In a large meta-analysis of epidemiological studies, there was an increased risk in smokers, both present and previous, compared with non-smokers. The risk increased both with the number of cigarettes smoked and the duration of smoking (151). Although statistically significant differences were found, the odds ratios were rather low. Since engagement in tobacco smoking is rapidly declining, however, this factor will be of less importance in the future.

#### Hereditary factors

There are some hereditary types of gastric cancer. The case of a Spanish family whose members had a missense mutation in one of the genes coding for the proton pump supports the theory that gastrin and ECL cells play important roles in gastric carcinogenesis, and that ECL cell NETs are precursors of gastric cancer (125). Moreover, the gastric cancer here was initially classified as an adenocarcinoma, but was reclassified by us as a tumor with mixed NET and carcinoma with neuroendocrine expression (152). The case of this Spanish family demonstrates without doubt that long-term hypoacidity will lead to cancer and thus the risk of profound acid inhibition.

Hereditary gastric cancer of the diffuse type due to a missense mutation of the *CDH1* gene coding for E-cadherin is a more prevalent type of genetic gastric cancer (153). In this context it should be mentioned that E-cadherin expression in ECL cells was not found by immunocyte/histochemistry, indicating that this cell type could be prone to invasion and metastasis (56). There are many very rare hereditary types of gastric cancer, which were discussed in a recent review (154) and which we will not comment on further in this paper.

#### Prophylaxis and therapy

With the knowledge of the main cause, *H. pylori*, and the most important pathogenetic factor, gastrin, the conditions for prophylaxis and treatment should be good. Most *H. pylori* infections occur early in life (24), and gastric cancer is most often diagnosed in old age (155). Previously, when the determination of basal and maximal gastric acid secretion was commonly conducted in clinical settings, it was accepted that gastric acid secretion declined with age. After the recognition of *H. pylori* as the dominant cause of gastritis (5), however, it was shown that the level of acid secretion declined with age only in individuals with *H. pylori* gastritis and was preserved in non-infected subjects (156). Due to the inclusion of *H. pylori*-positive individuals in control groups when determining the normal gastrin value at the time of establishing gastrin immunoassays, normal gastrin values have hitherto been too high (102, 112). The gastric acid secretion level declined with an increasing degree of oxyntic atrophic gastritis in the *H. pylori-*infected individuals (156). Whether or not the increase in blood gastrin above the new normal level based upon non-*H. pylori*-infected individuals can be used as a sensitive marker of early oxyntic atrophy has not been shown, but it seems logical that this would be the case. The description of *H. pylori* gastritis predisposing to gastric cancer only after having induced oxyntic atrophy (20) shows that *H. pylori* eradication should be carried out before this stage. The so-called “GastroPanel” including pepsinogen I and II and gastrin 17 did not report that gastrin (using the old normal value) was useful, but the ratio of pepsinogen I to pepsinogen II was reduced in a group of patients with dyspepsia (157). The prevalence of atrophic gastritis in *H. pylori*-infected children and young adults was reported to rise from 9.2% in children to 66.9% in young adults (aged between 18 and 40 years) (158). Thus, it seems rational to eradicate *H. pylori* in teenagers and young adults. Since the carcinogenic process continues after *H. pylori* eradication in individuals with oxyntic atrophy (34), presumably due to hypergastrinemia, it would be logical to treat such individuals with a gastrin antagonist such as netazepide, a substance with few, if any, side effects, and which has an effect on ECL cell neoplasms (88). Since gastrin is involved in carcinogenesis due to autoimmune gastritis and long-term profound acid inhibition, the use of netazepide is also a rational approach in these conditions (**Table 2**). Gastric cancer removal, either by surgery or endoscopically, is, of course, the cornerstone of any treatment. When curative surgery is not possible, a gastrin antagonist may be of value, at least in those patients with cancers expressing the gastrin receptor (91). Furthermore, the long-term drug-induced profound inhibition of acid secretion should be avoided. **Table 2** summarizes prophylaxis.

**Table 2:** Rational prophylaxis of gastric cancer in individuals with *H. pylori* infection or with autoimmune gastritis.

Test for *Helicobacter Pylori* (Hp) Antibodies	Treatment options	
Testing young at age about 20 years	Hp eradication	−
Before starting long-term PPI treatment All Hp positive adults	Hp eradication Hp eradication	− −
Those with oxyntic atrophy without Hp	−	Upper endoscopy/gastrin antagonist

(Reproduced with slight modifications with permission by the publisher from Cancers (Basel) 2020, 12, doi:10.3390/cancers12113477 (159)).

## Conclusion

The etiology and pathogenesis of gastric cancer are well known, and better known than for most other cancers. The central role of *H. pylori* (a bacterium) in gastric cancer is unique, although its effect (via inducing oxyntic atrophy leading to hypoacidity and hypergastrinemia) is indirect. Gastric carcinomas were classified in accordance with Lauren’s criteria (8) based on morphology as adenocarcinomas of intestinal and diffuse types depending on whether or not the presence of glandular structures was detected. This classification represents an important difference since the two types do not transform into the other, and the decline in prevalence seen in the last few decades is selective for the intestinal type. The diffuse type was initially classified among adenocarcinomas because PAS positivity was thought to represent mucin and thus exocrine cells. However, PAS has an affinity for glycoproteins in general. Many of the diffuse types express neuroendocrine and, more specifically, ECL cell markers, suggesting that these cancers develop from ECL cells, which are the target cells of gastrin.

Hypergastrinemia due to atrophic oxyntic gastritis also predisposes affected individuals to gastric cancer of the intestinal type, presumably originating from stem cells stimulated directly or indirectly (Reg protein from ECL cells) by gastrin. Moreover, all conditions resulting in gastric hypoacidity, including autoimmune gastritis, *H. pylori* gastritis, and profound acid inhibition due to PPIs will necessarily predispose affected individuals to gastric cancer. Since carcinogenesis most often is a slow process, a latency of decades must be expected. The central role of gastrin in gastric carcinogenesis provides an opportunity to prevent many gastric cancers. This may be achieved by the eradication of *H. pylori* before the occurrence of oxyntic atrophy, by avoiding the long-term use of drugs with profound inhibition of gastric acid secretion and by using gastrin antagonists when they become available, in those with autoimmune gastritis and in those with oxyntic atrophy due to previous *H. pylori* infection. Taking these steps will hopefully mean that gastric cancer becomes a rare disease.

## Author contributions

HW’s contribution has been central to the studies on gastric physiology and pathophysiology and gastric pathology. PM’s contribution has also been central to the pathology studies upon which this manuscript is based. HW took the initiative to draft this manuscript, but both authors have contributed to the writing process. All authors contributed to the article and approved the submitted version.
